# Chemical glycosylation of cytochrome c improves physical and chemical protein stability

**DOI:** 10.1186/1471-2091-15-16

**Published:** 2014-08-06

**Authors:** Yamixa Delgado, Moraima Morales-Cruz, José Hernández-Román, Yashira Martínez, Kai Griebenow

**Affiliations:** 1 Department of Biology, University of Puerto Rico, Río Piedras Campus, P.O. Box 70377, San Juan, Puerto Rico 00931-3346, USA; 2 Department of Chemistry, University of Puerto Rico, Río Piedras Campus, P.O. Box 70377, San Juan, Puerto Rico 00931-3346, USA

**Keywords:** Apoptosis, Chemical glycosylation, Drug delivery, Pharmaceutical protein, Protein formulation, Protein stability

## Abstract

**Background:**

Cytochrome c (Cyt c) is an apoptosis-initiating protein when released into the cytoplasm of eukaryotic cells and therefore a possible cancer drug candidate. Although proteins have been increasingly important as pharmaceutical agents, their chemical and physical instability during production, storage, and delivery remains a problem. Chemical glycosylation has been devised as a method to increase protein stability and thus enhance their long-lasting bioavailability.

**Results:**

Three different molecular weight glycans (lactose and two dextrans with 1 kD and 10 kD) were chemically coupled to surface exposed Cyt c lysine (Lys) residues using succinimidyl chemistry *via* amide bonds. Five neo-glycoconjugates were synthesized, Lac_4_-Cyt-c, Lac_9_-Cyt-c, Dex_5_(10kD)-Cyt-c, Dex_8_(10kD)-Cyt-c, and Dex_3_(1kD)-Cyt-c. Subsequently, we investigated glycoconjugate structure, activity, and stability. Circular dichroism (CD) spectra demonstrated that Cyt c glycosylation did not cause significant changes to the secondary structure, while high glycosylation levels caused some minor tertiary structure perturbations. Functionality of the Cyt c glycoconjugates was determined by performing cell-free caspase 3 and caspase 9 induction assays and by measuring the peroxidase-like pseudo enzyme activity. The glycoconjugates showed ≥94% residual enzyme activity and 86 ± 3 to 95 ± 1% relative caspase 3 activation compared to non-modified Cyt c. Caspase 9 activation by the glycoconjugates was with 92 ± 7% to 96 ± 4% within the error the same as the caspase 3 activation. There were no major changes in Cyt c activity upon glycosylation. Incubation of Dex_3_(1 kD)-Cyt c with mercaptoethanol caused significant loss in the tertiary structure and a drop in caspase 3 and 9 activation to only 24 ± 8% and 26 ± 6%, respectively. This demonstrates that tertiary structure intactness of Cyt c was essential for apoptosis induction. Furthermore, glycosylation protected Cyt c from detrimental effects by some stresses (i.e., elevated temperature and humidity) and from proteolytic degradation. In addition, non-modified Cyt c was more susceptible to denaturation by a water-organic solvent interface than its glycoconjugates, important for the formulation in polymers.

**Conclusion:**

The results demonstrate that chemical glycosylation is a potentially valuable method to increase Cyt c stability during formulation and storage and potentially during its application after administration.

## Background

Development of efficient cancer treatments is a top priority in health related research. One of the most common available options, in particular to treat advanced stages or as post-operation treatment, is chemotherapy which still today mostly employs extremely cytotoxic drugs that kill metabolically active cells. Such drugs include alkylating agents (e.g., cis-platin), anti-metabolites (e.g., 5-fluor uracil, gemcitabine), anti-microtubule agents (e.g., paclitaxel), and topoisomerase inhibitors (e.g., doxorubicin). Unfortunately, all these drugs have low tumor specificity and a frequently narrow therapeutic index thus producing significant unwanted side effects [1,2]. This situation has resulted in an intense search for cancer drugs that are more tumor-targeted and less toxic to normal cells [3-11]. As the result, a set of tumor-targeted nanomedicines is currently being studied that exploit hallmarks of cancer for reviews see references [12-14]. Targeting mechanisms include coupling of cytotoxic agents to ligands, antibodies, analogs targeting overexpressed receptors, formulation as nanoparticles to exploit the enhanced permeability and retention (EPR) effect, drugs specifically targeting enzymes involved in ontogenesis, among others [15-20]. Drug molecules involved in this area include small molecules, peptides, proteins, RNA molecules, and even complete oncolytic viruses [21-23].

Proteins are a potentially attractive class of drugs because they can be used to exploit the hallmarks of cancer by targeting specific events. One hallmark is that in cancer cells, particularly cancer stem cells, apoptosis is frequently disabled [22,24]. Delivery of a molecule to the cell switching apoptosis back on is therefore a potential treatment strategy. Cyt c is exactly such a molecule. It is a small mitochondrial heme protein involved in the intrinsic apoptotic pathway [24]. Severe DNA damage in cells and other events such as increase in P_53_, oxidative stress, and hypoxia lead to the activation of the intrinsic apoptotic pathway, which involves formation of pores in the mitochondrial membrane by BAX, BAK, and SMAC [25,26]. The BH3-only domain proteins directly bind and activate mitochondrial-localized BAK and BAX, triggering BAK/BAX oligomerization and inhibition of BCL-XL proteins [27,28]. The resulting depolarization of the mitochondrial membrane induces the release of Cyt c and other apoptotic factors into the cytoplasm [29]. Cyt c then interacts with the protein Apaf-1 to form the apoptosome [24].

Apoptosis is absent in many cancer cells for multiple reasons including mutation of critically important elements upstream from the event involving Cyt c [30]. Thus, a valid strategy is the delivery of Cyt c directly to the cytosol of cancer cells to induce apoptosis and eradicate the tumor. Using cancer models this has been accomplished multiple times by delivering Cyt c from nanoparticles [30-32] but it is yet unclear whether such systems would be sufficiently effective to allow for cancer treatment under real conditions. For example, uptake of nanoparticles frequently involves receptor-mediated endocytosis and thus the protein is exposed to conditions of low pH and proteases in lysosomes prior to the necessary endosomal escape [33-35]. Furthermore, the interstitium of tumors is frequently rich in proteases that are detrimental to pharmaceutical proteins [12,36].

In addition to aforementioned specific reasons, development of effective protein formulations is a significant challenge in the biopharmaceutical industry due to the inherent chemical and physical instability of proteins during production and purification [37], formulation [38], storage [39,40], transportation [39,41], and administration [42]. Particularly challenging is the development of smart drug-delivery and release systems for protein drugs in this context, because they typically rely on immobilization of the drug in the carrier [43,44]. The immobilization frequently employs linkers with pH- or redox-sensitive bonds that are cleaved in the cell but many proteins are structurally sensitive to the linker attachment, as we have demonstrated recently for carbonic anhydrase [45] and also for Cyt c [46].

Improving the stability of pharmaceutical proteins under the many different aforementioned circumstances, in particular during delivery, likely requires covalent strategies. One current example of such strategies consists in modifying FDA approved protein drugs with poly(ethylene glycol) (PEG) to afford increased plasma life time and reduced immunogenicity [16,47]. However, PEGs have not been reported to significantly stabilize proteins thermodynamically [48]. Also, PEGs are not biodegradable and can in tendency accumulate in the body [49]. A possible biodegradable alternative to modification of proteins with PEG is the covalent modification with carbohydrates. Chemical as opposed to natural glycosylation has recently been used as a strategy to stabilize commercially available medicines (i.e., glycosylated erythropoietin alfa (ARANESP®) and glycosylated brain natriuretic peptide (NATRECOR®) [50,51].

We have in recent years introduced chemical modification to stabilize several model proteins. We have systematically investigated the structure, function, dynamics, and stability interrelationships in the neo-glycoconjugates obtained [52-56]. We found that glycosylation of many model proteins provides the advantages typically associated with PEGylation but we also found significantly increased thermodynamic stability [57]. In this work we set out to investigate in more detail the stability, functional, and structural consequences of chemical protein glycosylation using for the first time a cancer relevant pharmaceutical protein, namely, Cyt c. We are able to demonstrate that chemical glycosylation is a potentially very useful method to stabilize Cyt c in pharmaceutical applications.

## Results and Discussion

**Figure 1:**
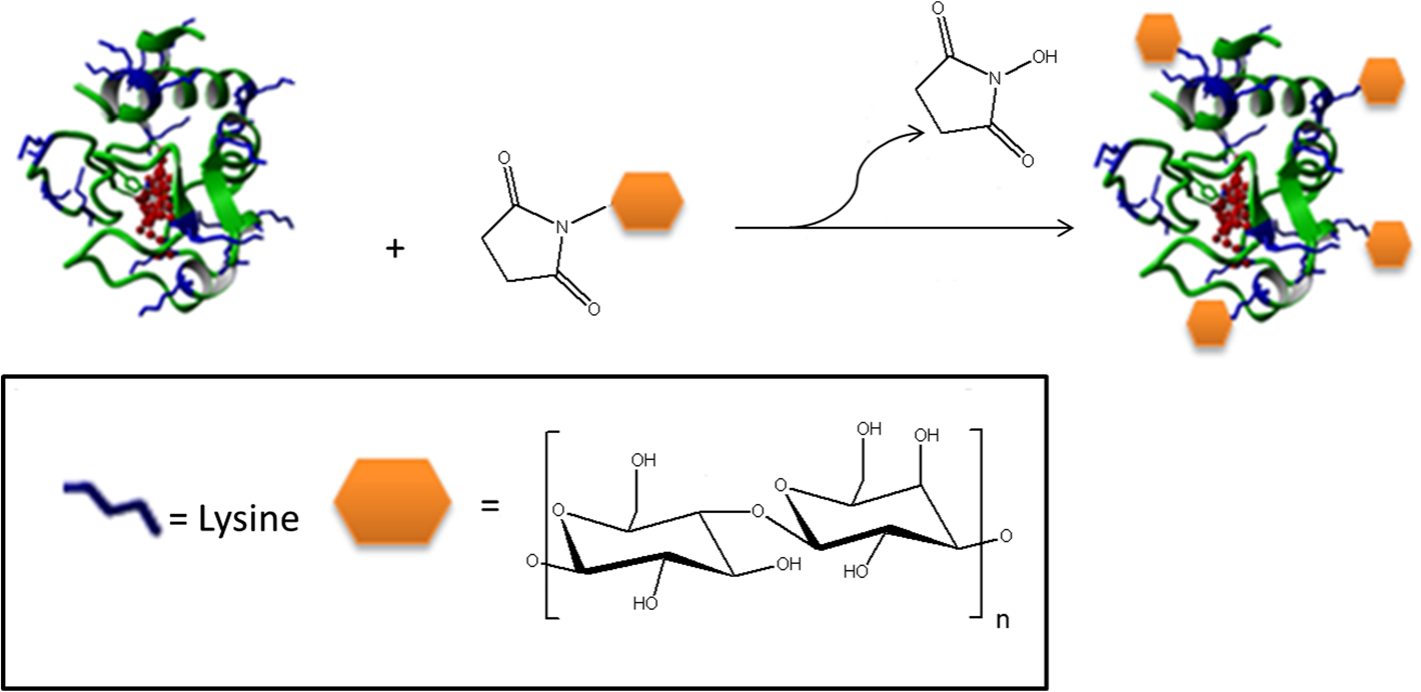
Representation of the chemical glycosylation of Cyt c using monofunctionally activated glycans.

Our strategy was to attach sugars via suitable linker chemistry to the ϵ-amino groups of lysine residues of Cyt c to establish various glycoconjugates. In order to vary the glycan size in our experiments, we chose dextran with molecular weights of 1 kD and 10 kD and lactose (0.5 kD) as glycans. The 10 kD dextran was first activated by reductive amination followed by modification with a homo-bifunctional linker to form amine reactive mono-(dextranamido)-mono-(succinimidyl)suberate (NHS-Dex, 10 kD) as described by us [52-55]. However, this did not work for the small dextran because the precipitation step failed. Thus, we used established 1-ethyl-3-[3-dimethylaminopropyl]carbodiimide hydrochloride (EDC)/N-hydroxysuccinimide (NHS) crosslinking chemistry with dextran hexanoic acid (Dex-COOH) to form NHS-Dex (1 kD). Activated lactose (NHS-Lac) is commercially available and was used as supplied. The amine and succinimidyl functionalization of the dextrans was followed by FTIR and NMR spectroscopy as described by us [52-55]. The activated sugars were then coupled to available amines of Cyt c (Figure 1) as previously described by us for various proteins [46,52-56].

The neo-glycoconjugates obtained were characterized (Table 1). Five different conjugates were obtained with the three different glycans. Throughout the manuscript we adopt the following nomenclature: Cyt c modified at 4 and 9 lysine residues with lactose was named Lac_4_-Cyt c and Lac_9_-Cyt c; Cyt c modified at 5 and 8 lysine residues with dextran of 10 kD were named Dex_5_(10kD)-Cyt c and Dex_8_(10kD)-Cyt c; and the glycoconjugate with 3 lysine residues modified with dextran of 1 kD was named Dex_3_(1 kD)-Cyt c. Glycosylation did not affect the pseudo-enzyme activity even for the most heavily glycosylated sample (Dex_8_(10kD)-Cyt c) in which there was a total of 80 kD of glycan attached to the roughly 13 kD protein. We can surmise that structure of Cyt c was likely not affected by binding of the sugars and that the substrate could still relatively freely diffuse to the active site even in the presence of a “sugar shell”.

**Table 1:** Functionality determination for Cyt c glycoconjugates

**Protein formulation**	**Glycosylation degree**^**#**^	**Residual activity**^**$ **^**(%)**	**Caspase 3 activation**^**% **^**(%)**	**Caspase 9 activation**^**% **^**(%)**	**Degradation rate, K**_**d**_^**& **^**(x 10**^**-2**^ **min**^**-1**^**)**
Cyt c	N/A	100	100	100	10.3 ± 0.1
Lac_4_-Cyt c	4.4 ± 0.4	94 ± 4	95 ± 1	95 ± 9	9.9 ± 0.2
Lac_9_-Cyt c	9.3 ± 0.2	97 ± 2	86 ± 3	93 ± 8	9.6 ± 0.1
Dex_3_(1 kD)-Cyt c	3.4 ± 0.9	98 ± 1	91 ± 2	96 ± 4	9.8 ± 0.2
Dex_5_(10kD)-Cyt c	5.2 ± 0.8	93 ± 4	89 ± 1	94 ± 6	9.5 ± 0.1
Dex_8_(10kD)-Cyt c	8.3 ± 0.4	95 ± 3	85 ± 2	92 ± 7	9.9 ± 0.3

^#^Glycosylation degree refers to the number of glycan modified Cyt c lysine residues as determined by 2,4,6-trinitrobenzene sulfonic acid (TNBSA) assay. ^$^The residual activity was calculated with respect to the specific activity of native Cyt c.^%^The caspase 3 and caspase 9 activation is with respect to the activation induced by non-modified Cyt c. The substrates used for caspase-3 and caspase-9 activation assay were 10 mM DEVD-pNA and 4 mM LEHD-pNA, respectively. ^&^The rate of degradation was calculated for Cyt c and Cyt c glycoconjugates after exposure to 1.5 mM H_2_O_2._ Each experiment was performed in triplicate, the values averaged, and the ± values are the calculated SD.

The use of Cyt c as an anti-cancer drug depends on the formation of the Apaf-1/Cyt c complex (apoptosome), which is responsible for the activation of the caspase cascade leading to apoptosis. Electrostatic interactions between positively charged lysine residues of Cyt c at the exposed heme edge and the WD-40 region of Apaf-1 are critically important to facilitate Cyt c-to-Apaf-1 binding [58,59]. Thus, it is imperative to preserve the Cyt c conformation subsequent to any chemical modification to assure that Cyt c is still able to bind to Apaf-1 and induce apoptosis. We performed a cell-free caspase 3 and 9 assays since Cyt c is a cell-membrane impermeable protein [30,31,60]. After the freeze and thaw cycles the lysate was centrifuged at 10,000 g to ensure mitochondrial removal. This is important to avoid false positives from mitochondrial leakage of Cyt c. Furthermore, we added the substrates to the lysates as controls and subtracted any small absorbance developing from the samples to which the external Cyt c or Cyt c glycoconjugates was added. The data obtained (Table 1) show that at most levels of glycosylation the bioactivity of Cyt c was still ca. 90% compared to non-modified Cyt c. This result is in thus far remarkable as there are lysine residues in the putative Cyt-c-Apaf-1 binding epitope. We have reported a similar finding albeit with Lac_4_-Cyt c and Cyt c-Lac_4_-SPDP for Cyt c activity on HeLa cells [46].

There are several potential explanations for this that need to be verified by site-directed glycosylation (currently ongoing in our laboratory). First, glycosylation could be inefficient for Lys residues in the Apaf-1 interaction epitope. We have highlighted why we believe this is happening in Additional file 1. Second, glycosylation could make Cyt c intrinsically more reactive and this could compensate for activity loss by glycosylation of residues in the interaction epitope. Third, glycosylation might not prevent Cyt c binding to Apaf 1 because ionic interactions are long-range. Regardless of the mechanistic reason, however, even highly glycosylated Cyt c was still remarkably active in activating the caspase cascade.

**Figure 2:**
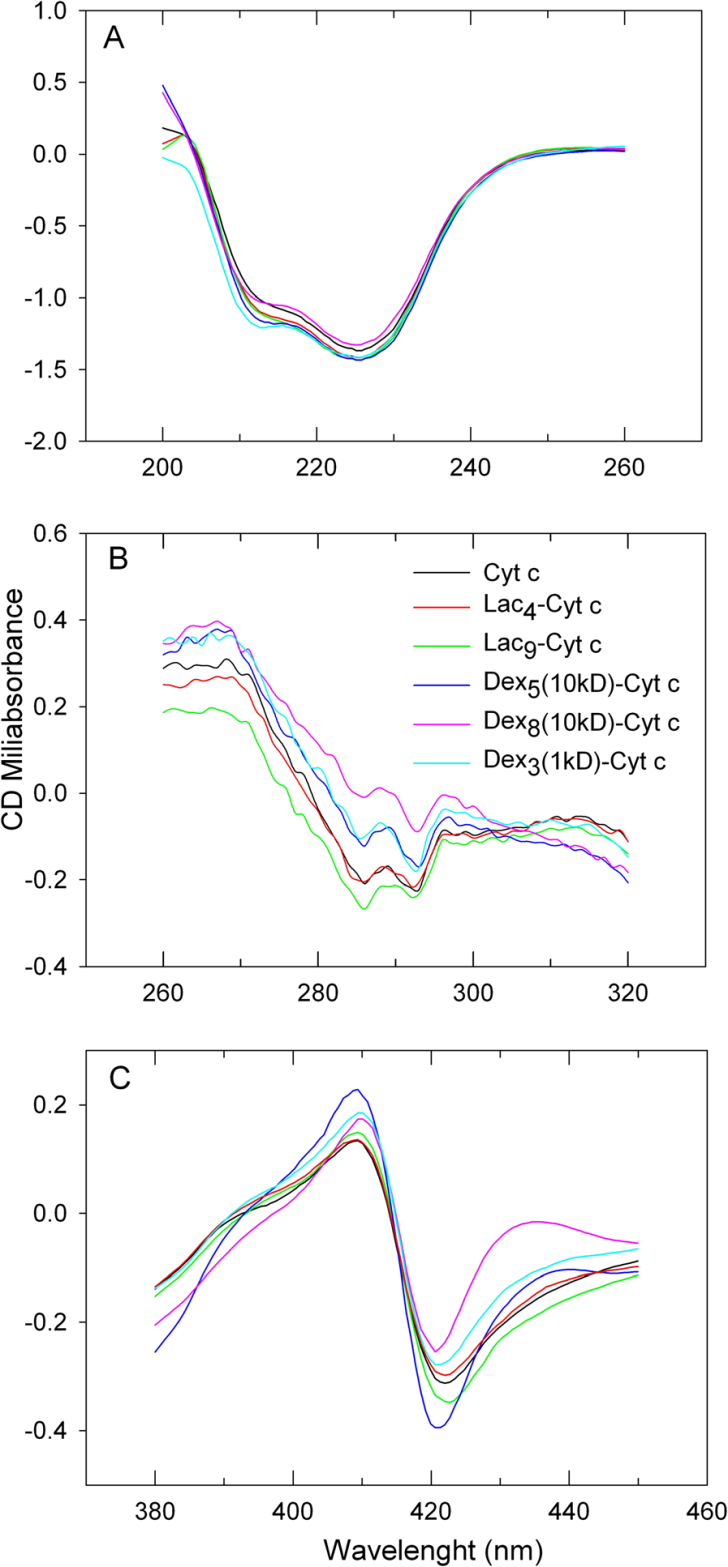
Far UV (A), near UV (B), and heme (C) region CD spectra of 0.6 mg/ml Cyt c and Cyt c glycoconjugates in 100 mM phosphate buffer at pH 7.4 and 20°C.

To investigate the structural consequences of glycan binding to Cyt c, we performed circular dichroism (CD) spectroscopy. Three spectral regions were investigated: the far-UV CD region from 200–280 nm which is sensitive to secondary structural perturbations, the near UV-CD region from 260–320 nm which is sensitive to tertiary structure perturbations, and the Soret region of heme absorbance from 380 to 460 nm which is sensitive to the heme environment and thus the structure of the heme binding pocket (Figure 2). In the far UV-CD region only minor spectral and thus secondary structural changes were observed. In contrast, spectral variations were evident when investigating the glycoconjugates in spectral regions that are sensitive to tertiary structural changes. The near UV-CD spectra of Cyt c and the lactose glycoconjugates were similar, while the dextran bioconjugates showed a reduced signal, which can be interpreted as being indicative of less tertiary structure (Figure 2B). Nevertheless, the CD spectra in the heme absorption region (Figure 2C) all showed a pronounced cotton effect, which is typical for a native heme environment. The Cotton effect, named after its discoverer Aimé Cotton (1869–1951), is the characteristic change in circular dichroism in the vicinity of an absorption band of a substance characterized by a positive and negative CD signal and a zero crossing at the absorption maximum. Structural changes in the heme-binding pocket lead to the disappearance of the cotton effect as reported by others and us for Cyt c [46,61]. This is in agreement with the activity data in Table 1, which show that all bioconjugates had a similar pseudo enzyme activity than Cyt c. We can surmise that for all bioconjugates the structure was native-like with the exception of a somewhat more lose tertiary structure packing in case of the dextran conjugates.

Next we proceeded to study the Cyt c stability in selected experiments. Chemical glycosylation has been shown to assist in overcoming chemical protein instability and provide protection against proteases [62-64]. This is highly relevant since it has been established that the tumor interstitial space presents significant trypsin-like protease activity and also oxidative stress [36]. Trypsin-like activity is particularly harmful to the integrity of basic proteins. The proteolytic degradation in tumors enables cell movement and the metastatic spread [31,65]. Potential improvement of Cyt c stability towards the tumor environment by glycosylation was tested by tryptic and chymotryptic assays.

**Figure 3:**
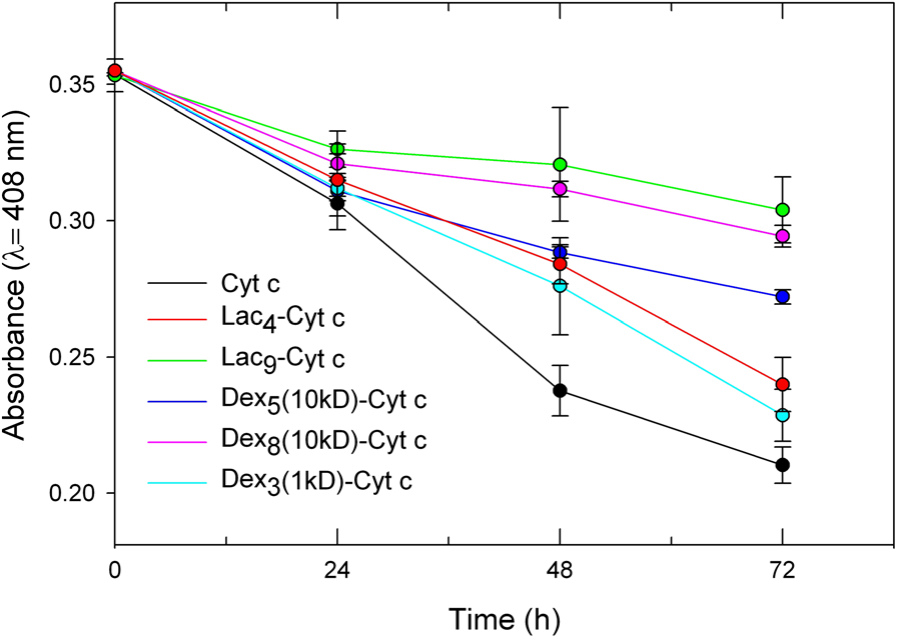
**Degradation of Cyt c and Cyt c glycoconjugates by 4 mg/ml trypsin at 37°C.** Each experiment was performed in triplicate, the values averaged, and the error bars are the calculated SD.

**Figure 4:**
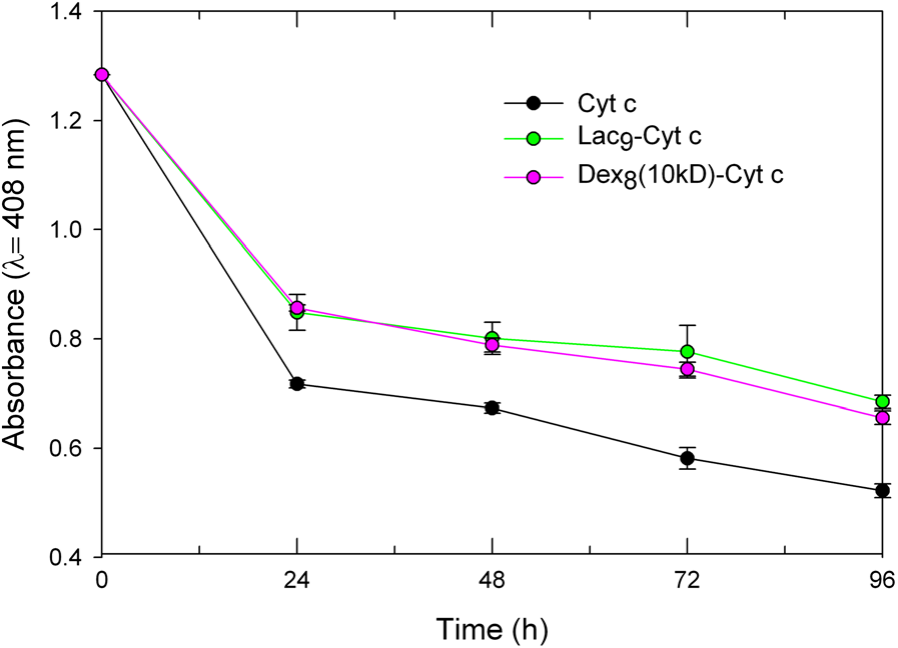
**Degradation of Cyt c and Cyt c glycoconjugates by 5 mg/ml α-chymotrypsin at 37°C.** Each experiment was performed in triplicate, the values averaged, and the error bars are the calculated SD.

The results of the tryptic digestion are shown in Figure 3. As anticipated, trypsin had a devastating effect on Cyt c integrity. Glycosylation stabilized the protein against proteolysis. It is evident that the amount of glycan bound to the protein is more significantly related to protection against proteolysis than the size of the bound glycan. For example, Lac_9_-Cyt c and Dex_8_(10kD)-Cyt c show similar protection from trypsin degradation even though there is about 20 times more glycan bound per weight in the dextran containing bioconjugate. This can be understood because trypsin cuts the peptide backbone next to Lys or Arg residues and the more Lys residues are chemically blocked, the lower the propensity for the proteolytic event to happen [66].To obtain a better idea of whether glycans in general protect Cyt c from proteolytic degradation by steric shielding, we also performed digestion with α-chymotrypsin, which cuts preferentially next to aromatic residues. We employed those samples for which glycosylation provided the best protection in the tryptic assay (Figure 3). It is evident that glycosylation protected Cyt c from proteolysis, albeit to a much lesser degree than in the tryptic assay (Figure 4). We can therefore conclude that glycosylation protects Cyt c from proteolytic degradation in general, but much better against those proteases having a binding pocket selective for Lys.

In summary, glycosylation should provide some protection against proteolytic degradation in the tumor interstitium and possibly after endocytosis of suitable constructs in lysosomes in agreement with our recent data using HeLa cells [46].

Cancer tissues frequently display an increased level of reactive oxygen species and oxidative stress as a result of persistent chronic inflammation [67]. The *in vivo* degradation of proteins by H_2_O_2_ interferes with normal cellular function, accelerates aging, and enhances metabolism of carcinogens [68,69]. Higher levels of free radicals also induce DNA mutations, which promote cancer progression and development of resistance [70]. To test whether glycosylation could protect the protein from oxidative stress, Cyt c was incubated with 200 μL of 1.5 mM of H_2_O_2_, which attacks the S-S bridge that covalently couples the heme group to apo Cyt c thus promoting unfolding [61,71,72]. The rate of degradation (K_d_) was determined from the decaying heme Soret absorbance (Table 1). No significant effect was found in this instance and chemical degradation by Cyt c with the small molecule H_2_O_2_ was not significantly slowed down. Therefore, it might be of value to protect Cyt c from the oxidative stress in tumor tissues by packaging it inside a delivery vehicle. The experiment also provides some insight into the effect of glycosylation on Cyt c structural dynamics or compactness of the folded state. The CD experiments revealed that Cyt c was not packed more densely as the result of glycosylation (Figure 2B) in contrast to what we have found for some other proteins [52-57]. This is not unexpected since Cyt c is intrinsically already very stable and not dynamic. The lack of an effect of sugars to stabilize Cyt c by reducing structural dynamics (molecular breathing) against the small molecule H_2_O_2_ is in agreement with the CD data.

**Figure 5:**
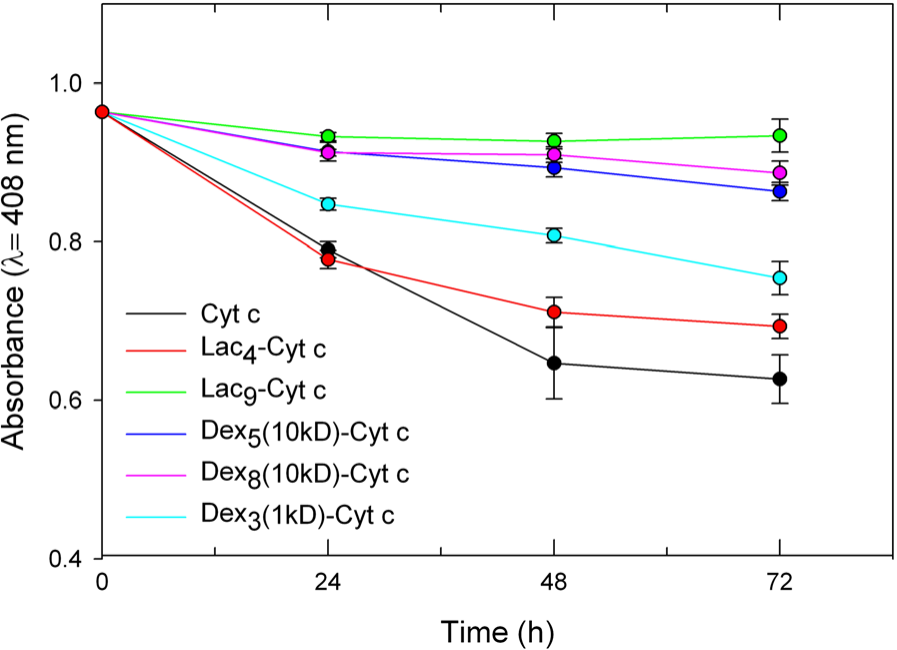
**The effect of 75% relative humidity on the structure of Cyt c and its glycoconjugates.** After incubation, the glycoconjugates were lyophilized and dissolved in 10 mM phosphate buffered saline at pH 7.3. Each experiment was performed in triplicate, the values averaged, and the error bars are the calculated SD.

**Figure 6:**
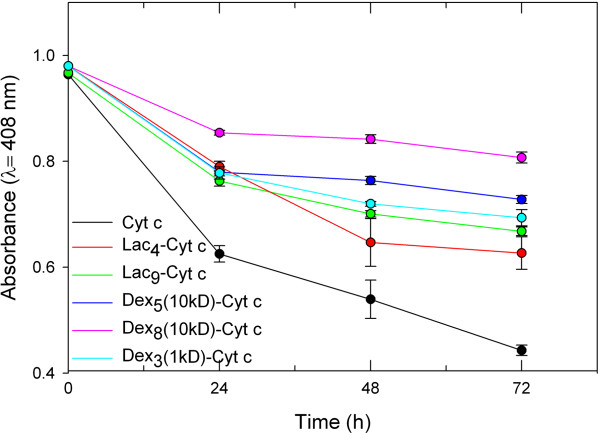
**The effect of incubation at 50°C on the structure of Cyt c.** After incubation, the glycoconjugates were lyophilized and dissolved in 10 mM phosphate buffered saline at pH 7.3. Each experiment was performed in triplicate, the values averaged, and the error bars are the calculated SD.

We have previously reported that chemical protein glycosylation was able to reduce moisture-induced aggregation of chymotrypsin under accelerated conditions [73], which is pharmaceutically relevant once vials or formulations of pharmaceutical proteins are exposed to moisture [74]. One has to keep in mind that this phenomenon is not only relevant to the exposure of lyophilized proteins to moisture upon opening sealed and dehydrated vials under atmospheric conditions, but also applies to the slow rehydration of dehydrated proteins inside of drug delivery systems [75]. Water frequently cannot penetrate the interior of drug delivery systems that are based on hydrophobic materials (e.g., poly(lactic-*co*-glycolic) acid) and encapsulated proteins are rehydrated slowly upon polymer swelling and degradation.Cyt c and Cyt c glycoconjugates were exposed to accelerated storage conditions of high humidity (75% relative humidity) (Figure 5) and high temperature (50°C) (Figure 6) for 72 h. It is evident that glycosylation substantially reduced the susceptibility of Cyt c to the heat and moisture stresses.

The stabilization of Cyt c towards moisture (Figure 5) was related to the amount and size of the glycan attached. The disaccharide lactose had only little effect on improving Cyt c stability at the lowest ratio. Increasing the amount of that glycan attached to Cyt c improved its stability. In contrast, dextran with a M_W_ of 10 kD practically completely prevented Cyt c degradation by moisture already when five molecules were bound.

Similarly, denaturation by heat was prevented the more efficiently the more glycan was bound to Cyt c and the larger the sugar entity (Figure 6). Our interpretation for the data is that the Cyt c surface was shielded by the glycans and that this reduced protein-protein interactions which can cause irreversible denaturation events. Regardless of the exact mechanism, it is clear that the Dex_8_(10kD)-Cyt c was the most stable preparation, in particular under elevated temperature conditions.

Finally, we also aimed at assessing the effect of exposure of Cyt c to an organic solvent interface. Such interfaces are common when preparing protein nanoparticles by precipitation [76] or upon protein encapsulation into hydrophobic polymers, e.g., by the water-in-oil-in-water double emulsion technique [77]. We selected one representative sample (Dex_3_(10 kD)-Cyt c) and two solvents previously employed in nanoparticle preparation following solvent precipitation protocols, i.e., acetone and acetonitrile [76]. Using various ratios, we found that 1:2 and 1:4 water-to-acetonitrile volume ratios were sufficient to induce Cyt c precipitation and result in stable suspensions. Next we tested the effect of the protein-solvent interface on the capacity of Cyt c to induce *in vitro* caspase 3 activation in a cell-free system (Table 2). Therapeutic use of Cyt c in cancer applications relies on the induction of apoptosis after the protein was delivered to the cytoplasm [24,26,58]. The results demonstrate that Cyt c suffered significant activity loss when precipitated with acetonitrile and acetone (Table 2). In contrast, glycosylation prevented inactivation completely for both solvents. We interpret the results as being indicative for the glycan preventing Cyt c – organic solvent interface interactions, which are typically initiated by hydrophobic protein surface patches and driven by entropic effects [78].

**Table 2:** **Results of the capability of Cyt c and Dex**_**3**_**(1 kD)-Cyt c to activate apoptosis after their exposure to water-organic solvent (o/w) interface conditions**

**Protein**	**Desolvating agent**	**Relative caspase 3 activation**^**# **^**(%)**
Cyt c	Acetonitrile	64 ± 8
	Acetone	51 ± 2
Dex_3_(1 kD)-Cyt c	Acetonitrile	100 ± 4
	Acetone	100 ± 3

^#^The relative activity was calculated with respect to the caspase 3 activation induced by Cyt c/Apaf-1 complex.

**Figure 7:**
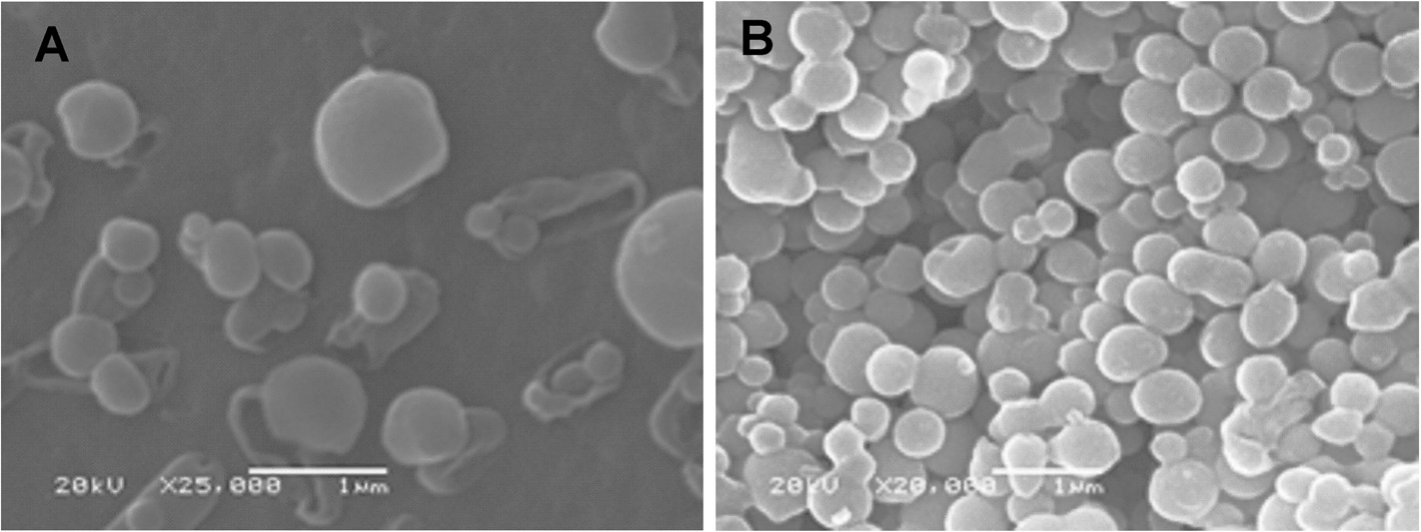
**Scanning electron microscopy (SEM) images of lyophilized Dex**_**3**_**(1 kD)-Cyt c after the precipitation via solvent displacement with (A) acetonitrile and (B) acetone as desolvating agent.**

While the Cyt c functionality upon contact with the two organic solvents was maintained regardless of the desolvating agent used, SEM images show differences in the particle properties after the precipitation process (Figure 7). SEM images of Dex_3_(1kD)-Cyt c show that the powder particles obtained with both solvents had a spherical shape and that the particle size was in the nanometer range. However, particles obtained by using acetone were smaller and more homogeneous than those obtained with acetonitrile. For drug delivery applications using the EPR effect a diameter of <400 nm and good homogeneity are desirable [19].

From our data we can surmise that chemical Cyt c glycosylation is a very useful approach to protect the protein from various stresses that are typically associated with pharmaceutical production processes and the delivery. Even though we have not conducted detailed mechanistic studies in this work, the data are consistent with the glycans providing protection in an amount and size dependent manner. The larger the glycan and the more attached to Cyt c, the better the protection. It is likely that the protection afforded is due to a combination of effects, i.e., restriction of the conformational space, reduction of solvent accessible area, and avoiding protein-solvent hydrogen bonds, as previously suggested by us [52-57] and others [49,62-64].

## Conclusions

Protein instability is a major factor limiting their use in pharmaceutical applications. Herein we explored how the covalent attachment of lactose and dextran via amine-directed linker chemistry affects the stability of Cyt c. Cyt c is a protein with good potential in cancer applications since it can be utilized to induce apoptosis upon intracellular delivery. We show that glycosylation does not negatively affect the capability of Cyt c to induce apoptosis. Furthermore, glycosylation improves the stability of Cyt c towards many stress factors relevant to the formulation, storage, and delivery of the protein into the cancer tumor microenvironment. We conclude that glycosylation should be a very useful method for improving Cyt c properties in cancer and other pharmaceutical applications.

## Methods

### Chemicals

Cytochrome c from equine heart (EC 232-700-9), dextran from *L. mesenteroides* (EC 232–6775, average M_w_ = 9–11 kD), trypsin from porcine pancreas, α-chymotrypsin type II from bovine pancreas, caspase 3 substrate (DEVD-pNA), caspase 9 substrate (LEHD-pNa), disuccinimidyl suberate linker, protease inhibitor cocktail, 2,2’-azino-bis(3-ethylbenzothiazoline-6-sulphonic acid) (ABTS), ammonium carbonate, dimethyl sulfoxide (DMSO), methylene chloride, acetone, and acetonitrile (HPLC grade) were from Sigma-Aldrich (St. Louis, MO). Mono-(lactosylamido)-mono-(succinimidyl) suberate (NHS-Lac) and dextran hexanoic acid (Dex-COOH, M_W_ = 1 kD) were from Carbomer (San Diego, CA). Cellulose ester dialysis membranes were from Spectrum (Rancho Dominguez, CA). EDC and NHS were from Proteochem (Denver, CO). A caspase-3 activity assay (CaspACE™ assay) was purchased from Promega (Madison, WI). HeLa cells, sera and culture media were purchased from the American Type Culture Collection (Manassas, VA). All other chemicals were from various commercial suppliers and were of analytical grade.

### Amine-reactive functionalization of 10 kD dextran

The synthesis of amine-reactive glycans was performed as previously established [52,78-80]. Synthesis of mono-(dextranamido)-mono-(succinimidyl) suberate (NHS-Dex, 10 kD) consisted of selective amination followed by succinnylation. Dextran (10 kD) (10.0 g, 1.0 mmol) and ammonium carbonate (2.0 g, 21.74 mmol) in nanopure water (50.0 ml) were stirred at 10°C for five days. Afterwards ammonium carbonate was removed by dialysis (M_W_ cut-off 100 D) for 48 h and the solution was lyophilized to afford 1-amino-dextran. To achieve dextran succinnylation, 1-hydroxy benzotriazole (0.675 g, 5.0 mmol) and disuccinimidyl suberate (1.841 g, 5.0 mmol) were dissolved in DMSO (60 ml) and heated to 80°C for 5 min. After cooling, 1-amino-dextran (5.0 g, 0.5 mmol) was added and the reaction maintained at 20°C for 24 h. The product was precipitated by addition of CH_2_Cl_2_ (200 ml) pelleted by centrifugation (1,500 rpm) at 4°C for 15 min. The resulting white precipitate was lyophilized to afford NHS-Dex (10 kD) in quantitative yield.

### Amine-reactive functionalization of Dex-COOH

Dex-COOH is chemically reactive to carbodiimides. Thus, EDC/NHS crosslinking chemistry was used to form the amine reactive NHS-Dex 1 kD to directly couple it to primary amines of Cyt c as described [81]. Briefly, 1 mg/ml of Dex-COOH in 2- (N-morpholino) ethanesulfonic acid (MES) buffer was reacted with 2 mM of EDC and 4 mM of NHS for 20 minutes at room temperature. Immediately, the glycosylation reaction was started for Dex (1kD)-Cyt c glycoconjugate.

### Glycosylation of Cyt c

Cyt c glycoconjugates were prepared by chemical glycosylation with succinimidyl-activated glycans (NHS-Dex(10 kD), NHS-Dex(1 kD) and NHS-Lac) as described by us in detail previously [53]. Briefly, 100 mg of Cyt c was dissolved in 20 ml of 100 mM phosphate buffer with 20 mM NaCl at pH 7.4. To achieve glycoconjugates with two levels of glycosylation, Cyt c solutions were reacted with an excess of 5 and 10 moles of NHS-Lac, an excess of 8 and 15 moles of NHS-Dex(10 kD) and an excess of 5 moles of NHS-Dex(1 kD). The reaction was performed for 1 h at room temperature under gentle stirring. Unbound glycans were removed by dialyzing thrice against nanopure water at 4°C for 48 h. After the dialysis, all the glycoconjugates were lyophilized for 48 h and then stored at -20°C. The extent of modification was determined by TNBSA colorimetric assay as described [82,83].

### Circular dichroism (CD) spectroscopy

CD spectra were recorded using an OLIS DSM-10 UV–vis CD spectrometer at 21°C. Cyt c and Cyt c glycoconjugates were dissolved in 20 mM PBS at pH 7.4. CD spectra were acquired from 200 to 260 nm (secondary structure), 260 to 320 nm (tertiary structure) and 380 to 450 nm (Soret region) at a concentration of 0.6 mg/ml using quartz cuvettes of 0.2 to 10 mm path lengths. Each spectrum was obtained by averaging six scans. Spectra of buffer blanks were measured prior to the samples and subtracted digitally using the software supplied with the instrument.

### Peroxidase pseudo-activity assay

The peroxidase-like activity of Cyt c was measured as described [84]. Briefly, the reaction was followed photometrically at 415 nm using 0.25 ml of 0.01 mg/ml Cyt c or Cyt c glycoconjugates, 0.2 ml of 300 mM H_2_O_2_, and 0.55 ml of 0.05 mM ABTS in 20 mM potassium phosphate buffer at pH 7. In all cases the specific activity (mM of ABTS^+∙^x min^-1^ × mg^-1^ Cyt c) was calculated. The activity was obtained by plotting the time-dependent absorbance changes *vs.* time. The linear portions of the graphs at less than 10% substrate conversion were used to obtain the initial velocities (V_0_). The experiments were performed in triplicate, the results averaged and the standard deviations calculated.

### Moisture and temperature-induced structural instability

The study of the effect of moisture and heat on Cyt c integrity during storage was performed as described [71,85]. Briefly, 0.60 mg/ml of Cyt c and Cyt c glycoconjugates in PBS at pH 7.4 (liquid-phase formulation) were exposed to accelerated stress storage conditions. To study moisture-induced instability, Cyt c and its glycoconjugates were incubated in a desiccator over NaCl salt slush at 20°C (which corresponds to 75% relative humidity) for 24, 48, and 72 h. To study heat- induced instability of Cyt c, the samples were incubated in a heat chamber at 50°C for 24, 48, and 72 h. After the desired length of time, the incubated protein samples were removed followed by 20 min of gentle stirring to ensure dissolution of the samples. The degradation of Cyt c samples was monitored at 408 nm [61,71,72]. Error bars in the figure are the calculated standard deviations (SD).

### Oxidative stress assay

The rate of Cyt c degradation upon exposure to oxidative stress was monitored as previously described [61,71]. In brief, 200 μl of 1.5 mM H_2_O_2_ was added to 1 ml of Cyt c and Cyt c glycoconjugates (0.2 mg/ml) in 20 mM PBS pH 7.4 at 21°C. Cyt c degradation was measured by monitoring the rate of the decrease in the Soret band absorbance at 408 nm after 10 min of incubation. The degradation rate constant (K_d_) was calculated from the residual absorption versus time using first order kinetics. The experiments were performed in triplicate, the results averaged and the SD calculated.

### Cell culture

HeLa cells were maintained in accordance with the ATCC protocol. Briefly, the cells were cultured in minimum essential medium (MEM) containing 1% L-glutamine, 10% fetal bovine serum (FBS), and 1% penicillin in a humidified incubator with 5% CO_2_ and 95% air at 37°C. All experiments were conducted before cells reached 25 passages. During each passage, cells were washed twice with PBS, detached using trypsin, and resuspended in supplemented MEM.

### Cell-free caspase 3 and caspase 9 activity assays

These assays were adopted from the literature [86]. In brief, HeLa cells were grown to 80% confluency and harvested as described above (cell culture method). For disruption the cells were suspended in 20 mM 4-(2-hydroxyethyl)-1-piperazineethanesulfonic acid (HEPES) buffer at pH 7.5, 10 mM KCl, 1.5 mM MgCl_2_, 1 mM sodium EDTA, 1 mM sodium EGTA, 1 mM DTT, 250 mM sucrose, and a protease cocktail inhibitors (serine, cysteine, aspartic acid, and metalloprotease inhibitors) (1×). The suspended cells were frozen in liquid N_2_ for 2 min and thawed in a 37°C water bath and the freeze/thaw cycle repeated thrice. The lysate was centrifuged at 10,000 × g for 20 min to remove the mitochondria. The protein concentration in the lysate was determined using the Bradford assay [87]. The cell-free reactions were performed in homogenizing buffer in a total volume of 100 μL. The reaction was initiated by adding 100 μg/ml of Cyt c or Cyt c glycoconjugates to freshly purified cytosol (3 mg/ml of proteins). The reaction was incubated at 37°C for 150 min. Later, 20 μl of the reaction mixtures was withdrawn and added to 78 μl of a mixture containing 100 mM HEPES at pH 7.5, 10% w/v sucrose, 0.1% w/v CHAPS (3-[(3-cholamido-propyl)-dimethylammonio]-1-propane-sulfonate), 10 mM DTT, and 2% v/v DMSO. This mixture was prepared for caspase 3 or caspase 9 assay in two different 96-well plates. Caspase 3 activity was measured using 2 μl of the substrate DEVD-pNA (10 mM) and caspase 9 activity using 2 μl of the substrate LEHD-pNa (4 mM). The plates were incubated overnight at room temperature and the absorbance at 405 nm was measured for both assays using a microplate reader (*Thermo* Scientific *Multiscan* FC). All measurements were performed in triplicate.

### Proteolytic assays

Protease degradation was performed as described by Reinhardt et al. [88]. In brief for tryptic degradation, 0.16 mg/ml of Cyt c or Cyt c glycoconjugates in 20 mM Tris–HCl at pH 8 were gently stirred for 20 min at 20°C. Then, 4 mg of trypsin was added to 1 ml of each sample and the mixture incubated at 37°C for 24, 48 and 72 h. For chymotryptic degradation, 0.79 mg/ml of Cyt c or Cyt c glycoconjugates in 20 mM Tris–HCl at pH 8 were gently stirred for 20 min at 20°C. Then, 5 mg of chymotrypsin was added to 1 ml of each sample and the mixture incubated at 37°C for 24, 48, 72 and 96 h. For both assays, Cyt c degradation was determined by measuring the absorbance at 408 nm after the incubation times.

### Protein precipitation by solvent displacement

A solvent displacement method similar to that described by us [76] was performed to precipitate Cyt c and obtain nanoparticles. Briefly, 5.0 mg of Cyt c was solvent-precipitated by adding 1.5 ml of acetonitrile to 1 ml of aqueous solution. To precipitate the Dex(1 kD)-Cyt c conjugate the concentration of the glycoconjugate was adjusted to 2.5 mg/ml and the volume ratios to precipitate it with acetonitrile and acetone were 1:2 and 1:1, respectively, in this case.

### Scanning electron microscopy (SEM)

SEM of lyophilized Cyt c and Dex-Cyt c after protein precipitation was performed using a JEOL 5800LV scanning electron microscope at 20 kV. The samples were coated with gold for 10 sec using a Denton Vacuum DV-502A.

## Abbreviations

ABTS: 2,2’-azino-bis(3-ethylbenzothiazoline-6-sulphonic acid); ATCC: American Type Culture Collection; Cyt c: cytochrome c; CD: circular dichroism; Dex: dextran; Dex-COOH: dextran hexanoic acid; EDC: ethyl-3-[3-dimethylamino-propyl]carboiimide hydrochloride; EPR: enhanced permeability and retention; FTIR: Fourier-transformed infrared; Lac: lactose; NHS: N-hydroxysuccinimide; NHS-Dex: mono-(dextranamido)-mono-(succinimidyl)suberate; NHS-Lac: mono-(lactoseamido)-mono-(succinimidyl)suberate; NMR: nuclear magnetic resonance; PEG: poly(ethylene glycol); SEM: scanning electron microscopy.

## Competing interest

The authors declare that they have no competing interest.

## Authors’ contribution

YD carried out all the experimental studies (except protein precipitation by solvent displacement and SEM), contributed to the experimental strategy, analyzed data, and drafted the manuscript. MMC carried out the protein precipitation by solvent displacement and SEM studies, analyzed data, and helped drafting the manuscript. JHR and YM helped in the experimental studies. KG conceived the study, participated in its design and coordination, and finalized the manuscript. All authors read and approved the final manuscript.

## Supplementary Material

Additional file 1 **The Additional file**1**contains two figures (Figure A1 and Figure A2) and discusses why we argue that chemical glycosylation did not diminish the capability of Cyt c to activate caspases 3 and 9.** Figure A1 shows the effect of β-mercaptoethanol exposure of Dex_3_(1 kD)-Cyt c on the far-UV CD, near-UV CD, and heme region CD spectra. Figure A2 shows the crystal structure (1HRC.pdb) of horse heart Cyt c including the solvent-exposed Lys residues. Click here for file
