# Supplementary material for: Chemical glycosylation of cytochrome c improves physical and chemical protein stability
Source: BMC Biochem. 2014 Aug 6;15:16. doi: 10.1186/1471-2091-15-16 (PMC4137108; doi:10.1186/1471-2091-15-16)
Supplement: Additional file 1 — The Additional file1contains two figures (Figure A1 and Figure A2) and discusses why we argue that chemical glycosylation did not diminish the capability of Cyt c to activate caspases 3 and 9. Figure A1 shows the effect of β-mercaptoethanol exposure of Dex3(1 kD)-Cyt c on the far-UV CD, near-UV CD, and heme region CD spectra. Figure A2 shows the crystal structure (1HRC.pdb) of horse heart Cyt c including the solvent-exposed Lys residues. [file 1471-2091-15-16-S1.docx]

**Additional Files to: Chemical Glycosylation of Cytochrome c Improves Physical and Chemical Protein Stability**

Yamixa Delgado^1^, Moraima Morales-Cruz^1^, José Hernández-Román^1^, Yashira Martínez^1^, and Kai Griebenow^1,2,^*

^1^Department of Biology, ^2^Department of Chemistry, University of Puerto Rico, Río Piedras Campus

After finding that Cyt c was able to activate caspase 3 and caspase 9 at even at high levels of glycosylation, we wanted to confirm that an intact Cyt c tertiary structure was necessary in this. We therefore used β-mercaptoethanol to perturb the tertiary structure of Cyt c [1]. We activated Dex-COOH using EDC/NHS crosslinking chemistry as described in the **Methods Section** to form the amine reactive NHS-Dex (1 kD) and coupled it to the Lys residues of Cyt c. After 1 h of completion of the glycan attachment reaction, the solution was incubated with 5 mM of β-mercaptoethanol for 10 min under constant stirring. The sample was dialyzed, lyophilized, and characterized as described in the **Methods Section** and had 3.1 ± 0.7 modified Lys residues. It was found that the exposure to β-mercaptoethanol reduced the caspase 3 activity from 91 ± 2% to 24 ± 8% and the caspase 9 activity from 96 ± 4% to 26 ± 6%.

We then investigated the secondary and tertiary structure and the heme absorption band of Dex_3_(1 kD)-Cyt c by CD spectroscopy (**Figure A1**). While the secondary structure was not affected as indicated by significant spectral changes in the far-UV CD, marked differences were found in the near UV-CD highlighting tertiary structure changes and in the heme band region. Thus, structural loss caused the decrease in caspase 3 and caspase 9 activation (**Table 1**).

The crystal structure of Cyt c is shown in **Figure A2** and the 19 solvent-exposed Lys residues are shown in lilac. The Lys patch (7, 25, 39, and 72) is composed of the residues reported to be necessary for the Apaf-1 binding. It is documented that the most important residue in this context is Lys 72 [2]. In addition, the heme group is very important for the electrostatic interaction [2-4]. The Cyt c/Apaf-1 binding is essentially characterized by electrostatic interactions of the positively charge Lys residues of Cyt c with negatively charged residues of Apaf-1. We performed the glycosylation reaction at neutral pH to preferentially modify the amino-terminus and the uncharged NH_2_ Lys in Cyt c. At neutral pH the majority of the Lys residues in Cyt c are positively charged and thus not very reactive. In addition, a past study of Cyt c identified four residues (Lys 25, 27, 86 and 87) to preferentially react with 1,4-benzoquinone [5]. The multiple basic residues that surround them influence the reactivity of these four Lys residues and they can be considered the most reactive Lys residues in Cyt c. This could explain why in our study glycosylated Cyt c still induces apoptosis even when it is highly modified.

**References**

1. Begg GE, Speicher DW: **Mass spectrometry detection and reduction of disulfide adducts between reducing agents and recombinant proteins with highly reactive cysteines.** *J Biomol Technol* 1999, **10:**17-20.
2. Yu T, Wang X, Purring-Koch C, Wei Y, McLendon GL: **A Mutational Epitope for Cytochrome *c* Binding to the Apoptosis Protease Activation Factor-1.** *J Biol Chem* 2001, **276:**13034-13038.
3. Kluck RM, Ellerby LM, Ellerby HM, Naiem S, Yaffe MP, Margoliash E, Bredesen D, Mauk AG, Sherman F, Newmeyer DD: **Determinants of cytochrome c pro-apoptotic activity. The role of lysine 72 trimethylation**. *J Biol Chem* 2000, **275:**16127-16133.
4. Olteanu A, Patel CN, Dedmon MM, Kennedy S, Linhoff MW, Minder CM, Potts PR, Deshmukh M, Pielak GJ: **Stability and apoptotic activity of recombinant human cytochrome c**. *Biochem Biophys Res Commun* 2003, **312:**733-740.
5. Labenski MT, Fisher AA, Lo H, Monks TJ, Lau SS: **Protein Electrophile-Binding Motifs: Lysine-Rich Proteins Are Preferential Targets of Quinones.** *Drug Metabol Disp* 2009, **37:**1211-1218.

**Figure A1.** Effect of β-mercaptoethanol exposure of Dex_3_(1 kD)-Cyt c on the far-UV CD, near-UV CD, and heme region CD spectra.

**
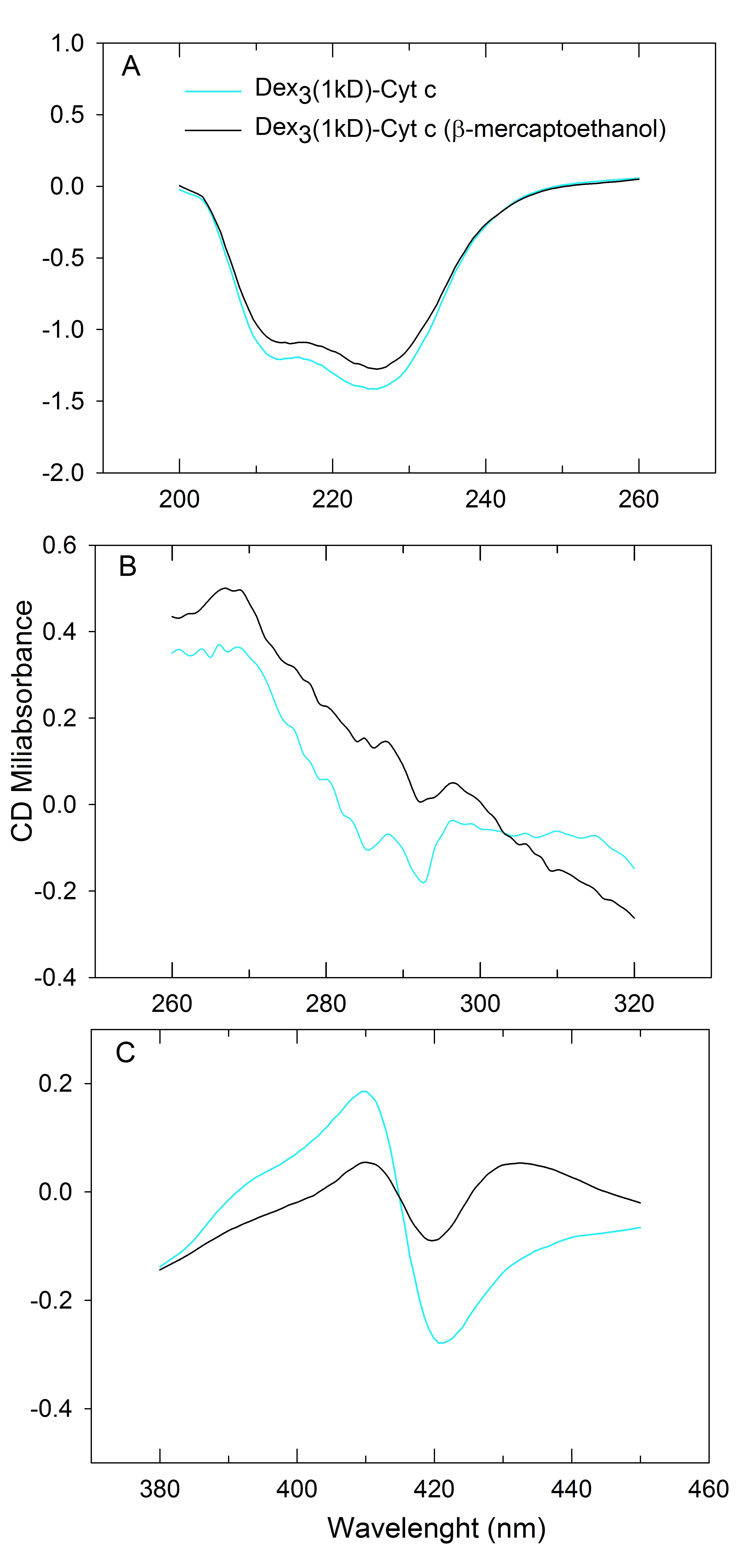
**

**Figure A2.** Crystal structure (1HRC.pdb) of horse heart Cyt c (**A**). Cyt c has 19 solvent-exposed Lys residues (5, 7, 8, 13, 22, 25, 27, 39, 53, 55, 60, 72, 73, 79, 86, 87, 88, 99, 100). The figure was generated using PyMol. Horse heart Cyt c sequence (**B**) from Uniprot P00004.

**
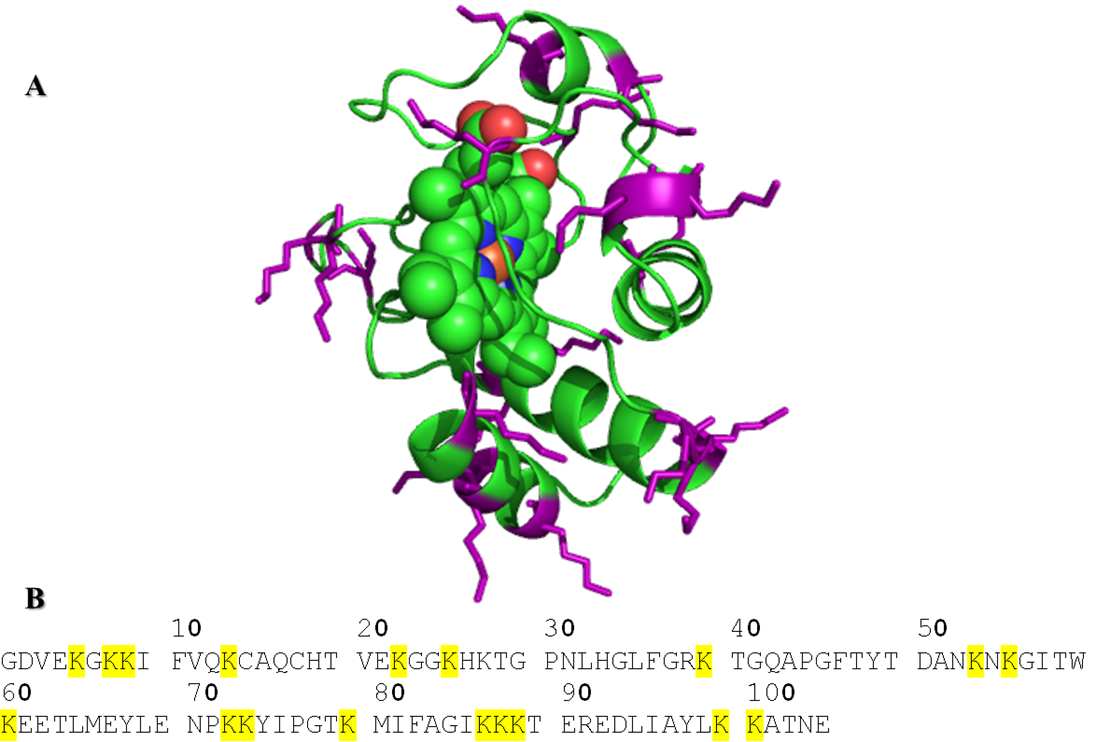
**
